# Role of circulating molecules in age-related cardiovascular and metabolic disorders

**DOI:** 10.1186/s41232-021-00187-2

**Published:** 2022-01-10

**Authors:** Yung Ting Hsiao, Ippei Shimizu, Yohko Yoshida, Tohru Minamino

**Affiliations:** 1 Department of Cardiovascular Biology and Medicine, Juntendo University Graduate School of Medicine, 2-1-1 Hongo, Bunkyo-ku, Tokyo, 113-8431 Japan; 2 Institute for Diseases of Old Age, Juntendo University Graduate School of Medicine, Tokyo, 113-8431 Japan; 3 Department of Advanced Senotherapeutics, Juntendo University Graduate School of Medicine, Tokyo, 113-8431 Japan; 4 Japan Agency for Medical Research and Development-Core Research for Evolutionary Medical Science and Technology (AMED-CREST), Japan Agency for Medical Research and Development, Tokyo, 100-0004 Japan

**Keywords:** Parabiosis, Aging, Rejuvenation, Circulating molecules, Cardiovascular diseases

## Abstract

Studies analyzing heterochronic parabiosis mice models showed that molecules in the blood of young mice rejuvenate aged mice. Therefore, blood-based therapies have become one of the therapeutic approaches to be considered for age-related diseases. Blood includes numerous biologically active molecules such as proteins, metabolites, hormones, miRNAs, etc. and accumulating evidence indicates some of these change their concentration with chronological aging or age-related disorders. The level of some circulating molecules showed a negative or positive correlation with all-cause mortality, cardiovascular events, or metabolic disorders. Through analyses of clinical/translation/basic research, some molecules were focused on as therapeutic targets. One approach is the supplementation of circulating anti-aging molecules. Favorable results in preclinical studies let some molecules to be tested in humans. These showed beneficial or neutral results, and some were inconsistent. Studies with rodents and humans indicate circulating molecules can be recognized as biomarkers or therapeutic targets mediating their pro-aging or anti-aging effects. Characterization of these molecules with aging, testing their biological effects, and finding mimetics of young systemic milieu continue to be an interesting and important research topic to be explored.

## Introduction

The level of molecules in circulation including proteins, metabolites, hormones, miRNAs change with chronological aging, and studies with parabiosis, blood exchange, and plasma transfer indicated these mediate the “anti-aging” or “pro-aging” effect in systemic organs. Heterochronic parabiosis, a parabiotic pairing between young and old mice, showed exposure to a young systemic environment leads to the rejuvenation of aged progenitor cells [1]. Exposure of aged mice to young blood resulted in rejuvenating synaptic plasticity and enhancement in cognitive function [2]. Factors found in young blood induced neurogenic rejuvenation of brains in aged mice and improved olfactory discrimination [3]. The heterochronic parabiosis model showed anti-geronic factors promote vascular rejuvenation in aged mice through attenuation of oxidative stress, and suppression of endothelial dysfunction [4]. Conversely, old blood was shown to induce alteration in mitochondrial structure and function in young mice [5]. Exposure of young mice to plasma from old mice induced reduction in synaptic plasticity, declined spatial learning and memory [6]. Single heterochronic blood exchange resulted in rapid inhibition of multiple tissues by old blood [7]. Studies indicate molecules in circulation increase or reduce with aging and affect systemic homeostasis. We would like to delineate these in this review article.

## Main text

### Role of circulating proteins or peptides in age-related disorders

Concentration of proteins or peptides in circulation changes with chronological aging, and this affects homeostasis in cardiovascular system [8, 9]. In this section, we will introduce these focusing on cardiovascular-metabolic disorders.

#### Growth differentiation factors

The aged heart is characterized by cardiomyocyte hypertrophy [10]. In non-obese, non-diabetic humans, the growth differentiation factor 11 (GDF11) level in circulation increased with aging and became highest in individuals aged 41–50. This declined thereafter and became lowest at age 71–80 [11]. GDF11 reversed age-related cardiomyocyte hypertrophy, indicating the role of this circulating protein in the suppression of cardiomyocyte hypertrophy [12]. GDF11 suppressed pathological myocardial remodeling in a murine diabetic cardiomyopathy model through the SIRT1 signaling pathway [13]. In vitro studies showed administration of GDF11 prevented hypertrophy in cultured neonatal rat ventricular myocytes treated with norepinephrine [14]. Another report showed this molecule mediated antiapoptotic effects in cardiomyocytes under hypoxic conditions through activation of autophagy [15]. GDF11 was also shown to improve endothelial dysfunction, inhibit endothelial apoptosis, and reduce atherosclerotic plaque in apolipoprotein E knockout (ApoE KO) mice [16]. Interestingly, a recent study showed cardiomyocyte-specific depletion of GDF11 (Myh6-Cre; Gdf11 floxed mice) did not result in cardiac hypertrophy but showed left ventricular dilatation [17]. It needs to be noted that in this paper, the level of GDF11 did not reduce in cardiac tissue possibly through the production of this molecule by non-cardiomyocytes, and some other animal models should be generated and studied to test the role of GDF11 in cardiomyocytes as authors mentioned in this paper. Issues to be considered would be the potential side effect of GDF11 in skeletal muscle. GDF11 inhibited muscle regeneration and reduced satellite cell expansion in mice [18]. In the hindlimb ischemia model or Duchenne muscular dystrophy model mice, inhibition of GDF11 resulted in the enhancement of muscle hypertrophy and grip strength [19]. This indicated GDF11 suppression (not activation) may become a therapeutic target for sarcopenia. Studies indicate GDF11 mediates beneficial roles in cardiac tissues and detrimental effects in skeletal muscle; however, we also need to note that a high level of GDF11 (5.0 mg/kg) caused severe body weight loss, cachexia, and death [20]. Another report concluded introduction of GDF11 (0.1 mg/kg) had no effect on cardiac structure or function [21]. Together with the exploration of optimized concentration, further studies are needed to test the therapeutic potential of this protein in cardio-vascular metabolic disorders.

GDF15 is a stress-responsive cytokine having multiple and possibly bidirectional roles in pathogenic conditions. Circulating GDF15 was reported to increase with aging in humans, and this inversely associated with active lifestyle [22], showed a positive correlation with signs of accelerated aging and all-cause mortality in aged individuals [23]. Elevation in GDF15 is associated with a reduction in muscle mass and strength in older patients at hospitalization [24]. GDF15 level predicted all-cause mortality in patients with heart failure [25]. The level of GDF15 in circulation was reported to predict 11-year mortality in older adults [26]. In patients with atrial fibrillation, GDF15 was a risk factor for major bleeding, stroke, and mortality [27]. GDF15 was also shown to be associated with various cardiovascular events in coronary heart diseases, heart failure, and atherosclerosis [28]. GDF15 was reported to have an equal diagnostic capability in heart failure with preserved ejection fraction (HFpEF) patients compared with N-terminal pro-brain natriuretic peptide (NT-proBNP) [29]. Heart failure with reduced ejection fraction (HFrEF) patients had a higher level of this molecule in circulation compared with heart failure with midrange ejection fraction (HFmrEF) [29]. Controversies exist in the role of GDF15 in pathological conditions. In the heart, GDF15 is considered to mediate anti-inflammatory, anti-oxidative, and anti-apoptotic effects and mediated cardioprotective effects [30]. GDF15 ameliorated cardiac injury in the ischemia-reperfusion model [31]. In this paper, the authors also showed GDF15 suppressed apoptosis in cultured cardiomyocytes subjected to ischemia-reperfusion [31]. Generation of myocardial infarction in the GDF15 knockout (KO) model led to an increase in cardiac rupture in the KO group [32]. Mechanistically, GDF15 suppressed the infiltration of polymorphonuclear leukocytes into the infarcted myocardium, thereby suppression of this molecule led to this rupture-prone cardiac phenotype after ligation of the coronary artery [32]. In vitro studies showed GDF15 increased phosphorylation of TGF-beta-activated kinase1 (TAK1) in C2C12 cells, and the TAK1 inhibitor led to a reduction in the marker of muscle atrophy [33]. Senescent endothelial cells secreted GDF15 [34] and activated the ROS-mediated p16 signaling pathway [35]. The role of GDF15 may be context dependent, and further studies are needed to explore whether modulation of GDF15 becomes a therapy in some specific conditions.

#### MMPs and their inhibitors

Matrix metalloproteinases (MMPs) belong to a large family with zinc-dependent enzyme activity and are able to degrade the extracellular matrix. The negative endogenous regulators are tissue inhibitors of metalloproteinases (TIMPs). This directly binds to the catalytic domain of MMPs and suppresses their activity. Among them, the roles of MMP-2, MMP-9, TIMP-1, and TIMP-2 are extensively studied in atherosclerotic vascular disorders. In atherosclerotic lesions, MMP-2 and MMP-9 are considered to enhance leukocyte infiltration, vascular smooth muscle cell (VSMC) migration, and plaque formation and make plaque susceptible to rupture [36–42]. The level of MMP-2 and MMP-9 in circulation was higher in acute coronary syndrome (ACS) patients compared with stable angina patients [43]. Patients with peripheral arterial disease or intermittent claudication and critical ischemia had a high level of MMP-2, MMP-9, and TIMP-1 in circulation, and no change was observed in TIMP-2 expression [37, 38, 44]. Abdominal aortic aneurysm patients also exhibited high MMP-9 expression in circulation [45]. In the acute phase of aortic dissection (within 24 h of symptom onset), the level of MMP-1 and TIMP-1 in circulation became high, but MMP-2 and MMP-9 were similar compared with healthy individuals [46]. With aging, serum MMP-3 and TIMP-1 increased; in contrast, MMP-10 and TIMP-2 are reduced in humans [47]. This suggested changes in MMP/TIMP balance develop with aging. Studies indicate the level of MMPs and TIMPs would become biomarkers for cardiovascular disorders; however, further studies are needed to test whether they become direct therapeutic targets (please also see the following review article [48]).

#### Inflammatory cytokines

Level of pro-inflammatory cytokines such as interleukin-6 (IL-6), IL-1β, tumor necrosis factor α (TNF-α), and C-reactive protein (CRP), molecules contributing to the enhancement of inflammation including soluble intercellular adhesion molecule-1 (ICAM-1), and vascular cell adhesion molecule-1 (VCAM-1) increase in circulation with chronological aging [49–52]. Chronic low-grade sterile inflammation associates with the production of reactive oxygen species (ROS), mitochondrial dysfunction, and cellular senescence and promotes pathologies in cardiovascular-metabolic disorders [8, 53]. The level of CRP in circulation is a most established marker for atherosclerotic vascular diseases [54–57]. A high CRP level in the blood was reported to be an independent risk factor for myocardial infarction (MI) [58] and predicted poor outcome in peripheral arterial disease (PAD) patients [54, 59]. CRP is a powerful tool for risk prediction but in general not considered a therapeutic target [60]. The serum concentration of IL-6 increases with age [52], and this cytokine is also recognized as a marker for cardiovascular disease. In humans, the elevated level of IL-6 is associated with higher cardiovascular mortality [61, 62]. Interestingly, IL-6 showed a more consistent and stronger predictive value than other inflammatory molecules in patients with PAD [8]. The level of TNF-α in circulation increased with aging [52], and the concentration of this cytokine in plasma showed a positive correlation with pro-atherogenic cytokine levels in atherosclerotic plaque of patients subjected to carotid surgery [63]. Plasma TNF-α elevated in post-myocardial infarction patients at increased risk for recurrent coronary events [64]. High circulating TNF-α is associated with increased mortality in patients with HFrEF or HFpEF [65]. TNF-α was reported to stimulate endothelial permeability, increase endothelial adhesion molecules, and promote inflammation in vessels [66, 67]. TNF-α was also shown to induce insulin resistance and inhibit endothelium-dependent vasodilation in humans [68]. The pacing-induced dog heart failure model showed suppression of TNF-α ameliorated cardiac dysfunction by improving mitochondrial function and inhibiting ROS production [69]. The therapeutic potential of TNF-α was tested in clinical studies. A TNF-α antagonist, adalimumab, suppressed inflammation in the ascending aorta and carotids and decreased CRP by 51% in psoriasis patients [70]. Adalimumab was also shown to improve endothelial function and arterial stiffness in patients with severe psoriasis [71]. In rheumatoid arthritis patients, another TNF-α antagonist, infliximab, improved endothelial function [72]. Infliximab was tested in HFrEF patients, and this study concluded infliximab did not improve the clinical condition of moderate-severe heart failure at 28 week follow up period [73]. In healthy individuals, aging was shown to increase IL-1β in circulation [52]. Canakinumab, an IL-1β neutralizing antibody, significantly reduced cardiovascular events together with reduction in circulating CRP and IL-6 at the follow-up [74]. In patients with prior myocardial infarction, canakinumab administration resulted in dose-dependent reduction in hospitalization for heart failure [75]. Residual inflammatory risk was reported to relate to both IL-18 and IL-6 after IL-1β inhibition, and IL-18 and IL-6 are considered to be important therapeutic targets [76]. Serum soluble VCAM-1 (sVCAM-1) but not soluble ICAM-1 (sICAM-1) was shown to have a positive correlation with aging [77]. Studies showed high expression of circulating sVCAM-1 [78, 79] or sICAM-1 [79, 80] in PAD patients. In preclinical studies testing mice, blocking ICAM-1 suppressed angiotensin II (AngII)-induced cardiac remodeling [81]. Further studies are needed to test whether targeting VCAM-1 and ICAM-1 becomes a therapy for age-related diseases.

#### Angiotensin II

Angiotensin II (Ang-II) is the main bioactive product of the renin–angiotensin–aldosterone system (RAAS), and contributes for the activation of Ang II type 1 receptor (AT1R) mediated pathogenic signaling [82]. RAAS is considered to become activated in vessels and cardiac tissues of humans and rodents with aging [83]. This leads to an increase in ROS level together with inflammation in these organs and enhanced tissue remodeling [84, 85]. Administration of an ACE inhibitor, enalapril, suppressed age-associated increases in cardiomyocyte apoptosis and mitochondrial ROS level [85]. The genetic model with AT1R depletion led to phenotype with longevity [86]. In a normotensive rat, the plasma Ang-II level was shown to increase with age [87], however, Ang-II was not shown to increase in plasma with aging in healthy human volunteers [88]. Considering RAAS activation develops with aging and promotes tissue remodeling in cardiovascular system, suppression of this pathway continues to be important targets in cardiovascular disorders.

#### Osteocalcin

Osteocalcin (OC) is a vitamin K-dependent protein which is produced by osteoblasts. This is secreted when insulin binds to osteoblasts in the presence of vitamin K, and can be recognized as a marker for bone remodeling [89, 90]. Level of OC in circulation shows interesting behavior with aging. Both in male and female individuals, this decreased with aging from 20 to 49 years old [91]. In male, no significant correlation was observed after 50 years old [91]. In female, after showing age-dependent decline till 49 years old, serum OC markedly increased at age 50 years, and thereafter again started to decrease with aging [91]. A systematic meta-analysis concluded an inverse associations of circulating OC with risk of atherosclerotic outcomes and cardiovascular disease (CVD) endpoints [92]. An increase in OC is associated with lower pulse wave velocity (PWV) in aged diabetic male patients but this was not observed in female patients [93, 94]. In mice, OC administration improved hippocampal-dependent memory [95], and exercise capacity [96]. Another study showed OC prevents age-related muscle loss in mice [97]. Total OC (tOC) have two primary forms, including undercarboxylated osteocalcin (ucOC) and carboxylated osteocalcin (cOC), with distinct functions in each form. Aging was shown to be associated with the U-shaped pattern for tOC, cOC, and ucOC levels. The ucOC/tOC ratio was higher; in contrast, the cOC/tOC ratio was lower in men with advanced age [98]. Some studies showed ucOC was a main bioactivity form of OC, contributing to the regulation in glucose homeostasis and energy metabolism [99, 100]. Another study showed ucOC improved microvascular insufficiency and myocardial dysfunction in a rat diabetic cardiomyopathy model [101]. However, the following studies have challenged these findings by showing no endocrine abnormalities in OC-deficient mice [102, 103]. In humans, higher ucOC is associated with lower pulse wave velocity (PWV), but administration of this molecule in rabbits did not result in alteration of vasoactivity of carotid arteries [104]. Focusing on the specific form of OCs, further studies are needed to test the therapeutic potential of these molecules in age-related diseases.

#### Meteorin-like

Meteorin-like is an adipokine, highly expressed in white adipose tissue. In patients with coronary artery disease (CAD), the level of Meteorin-like showed a negative correlation with the severity of CAD [105]. Some papers analyzing type 2 diabetic patients showed Meteorin-like elevated in circulation [105–109]. Other papers showed newly diagnosed type 2 diabetes patients had a significantly lower level of Meteorin-like in circulation [110, 111]. And recently published meta-analysis concluded no significant change in circulating Meteorin-like was observed in patients with diabetes [112]. Low serum Meteorin-like is associated with glucose intolerance, endothelial dysfunction, and atherosclerosis [105–108]. A study with rodents showed increasing Meteorin-like in circulation enhanced energy expenditure and improved glucose tolerance in mice [113]. This molecule also improved glucose tolerance in skeletal muscles [114]. Systemic depletion of Meteorin-like decreased blood HDL cholesterol and increased triglyceride in blood [115]. Doxorubicin-induced cardiotoxicity was suppressed with Meteorin-like through the activation of cAMP/PKA/SIRT1 signaling [116]. Meteorin-like also suppressed pancreatic β-cell apoptosis and promoted proliferation of this cell [117]. Meteorin-like was reported to enhance skeletal muscle repair, indicating this molecule has roles for muscle regeneration [118]. Preclinical studies indicate the protective role of this molecule in cardiovascular metabolic disorders and further studies are needed to analyze the level of this molecule in aging and test whether Meteorin-like can mediate favorable effects in humans.

#### Mesencephalic astrocyte-derived neurotrophic factor

Mesencephalic astrocyte-derived neurotrophic factor (MANF) is a secreted stress-response protein, and the level of this protein was reported to decline with aging in flies, mice, and humans [119]. MANF overexpression resulted in the extension of the life span in flies, and heterozygous depletion resulted in the progression of liver damage and fibrosis, and supplementation of this molecule improved age-related metabolic dysfunction [119]. Overexpression of MANF in the liver resulted in suppression of adipose inflammation, improved insulin sensitivity, and hepatic steatosis [120]. In contrast, liver-specific MANF depletion exacerbated obesity, insulin resistance, and hepatic steatosis [120]. MANF had critical roles for pancreatic β-cell proliferation [121] and protected human pancreatic beta cells against stress-induced cell death [122]. This molecule contributed to the protection of neuronal apoptosis in a rat model of intracerebral hemorrhage [123]. MANF suppressed ischemia-reperfusion mediated cell death in the heart [124]. A recent report showed MANF knockdown increased cardiac damage after ischemia-reperfusion in the heart, and this was reversed by MANF overexpression [125]. Further studies would be needed to test whether MANF administration mediates favorable effects in humans.

#### Fibroblast growth factor 21

Fibroblast growth factor 21 (FGF21) is a member of fibroblast growth factor (FGF), and circulating FGF21 is considered mainly produced in the liver. Patients with coronary artery disease exhibiting a high serum FGF21 level had a greater risk of developing major adverse cardiovascular events (MACEs) [126]. FGF21 increased in nonalcoholic fatty liver disease patients [127]. In healthy individuals, circulating FGF21 increased with aging [128]. Interestingly, FGF21 in circulation reduced with endurance exercise in the elderly [129], and the level of this protein was low in healthy centenarians [130]. Compared with young individuals (< 40 years), FGF21 was high in elderly individuals (> 70 years); however, FGF21 responsiveness in adipose tissue was similar, and this suggested the presence of FGF21-resistant state with aging [131]. FGF21 stimulates glucose uptake in adipocytes, and FGF21 transgenic mice became resistant to diet-induced obesity [132]. FGF21 administration ameliorated age-related metabolic disorders including insulin resistance, dyslipidemia, and obesity in rodents [132, 133]. FGF21 extended the lifespan in mice, and this was considered through blunting the growth hormone/insulin-like growth factor-1 signaling pathway in the liver [134]. In the aged mice, FGF21 was reported to prevent low-protein diet-induced renal inflammation [135] and promoted remyelination in the central nervous system [136]. To further test the role of FGF21, PEGylated FGF21 was administrated in humans with obesity and diabetes predisposed to fatty liver [137]. HbA1c showed no change, but metabolic parameters and fibrosis markers improved with administration of this compound [137]. Biological effects of a variant of FGF21, LY2405319, were tested in patients with obesity and diabetes [138]. LY2405319 treatment resulted in a significant improvement in dyslipidemia, and this was associated with favorable effects on body weight, fasting insulin, and adiponectin [138]. Another FGF21 mimetic, PF-05231023, was tested in humans by single intravenous injection to test safety and tolerability, and the study concluded this compound as safe [139]. Enhancement of FGF21-mediated signaling continues to be an interesting approach.

#### Activin A

Activin A belongs to a TGF-β superfamily and is known to mediate its biological effect through activin type II receptor (ActRII) signaling. Serum Activin A was reported to increase with aging [140]. This molecule was also reported to show dose-dependent association with metabolic syndrome [141] or diabetes and correlated positively with carotid intima-media thickness in prediabetes individuals [142]. Patients with diabetic kidney disease had a higher level of Activin A in circulation, and this showed a positive correlation with reduced kidney function [143]. Non-alcoholic steatohepatitis (NASH) patients were also reported to have high expression of this protein in circulation [144]. Mice aged 28 months had around a 3-fold increase in the Activin A level compared with 4-month-old mice, and this was considered to activate ActRII signaling in the aged heart [145]. They also showed the Activin A level in the blood increased with left ventricle pressure overload, and overexpression of this molecule induced cardiac dysfunction in young mice [145]. GDF11 is another ligand for ActRII contributing to the activation of this receptor. As already demonstrated, several reports showed the cardio-protective role of GDF11 in the suppression of age-related cardiomyocyte hypertrophy [12], or myocardial remodeling in diabetic mice [13]. In the same report by Roh et al., GDF11 was shown to activate ActRII signaling in the heart and reduced cardiac function [145]. The same group showed pharmacological suppression of ActRII ameliorated cardiac dysfunction in a murine left ventricle pressure overload model [145]. Anti-Activin A therapy was introduced in humans with a monoclonal antibody describing Garetosmab, and no critical safety issue was raised [146]. Further studies would show whether targeting Activin A would become a therapy for cardiovascular-metabolic diseases.

#### Insulin and insulin-like growth factor-1

Insulin or insulin-like growth factor-1 (IGF-1) binds to insulin receptor or IGF-1 receptor and mediate their biological effects through nearly identical downstream signaling. With aging, the circulating-insulin level in response to oral glucose load declined [147]. Aging is considered to be associated with relative insulin secretory defects, and together with the reduction in the clearance of this molecule, the level of insulin is considered similar between young and aged individuals [148]. The genetic model showed a reduction in circulating insulin by ~25% improved systemic insulin resistance and extended the lifespan in mice [149]. Homozygous depletion of insulin receptor in cardiomyocytes resulted in cardiac dysfunction in mice [150], but heterozygous depletion was cardioprotective in the murine model with left ventricle pressure overload [151]. Insulin resistance predicted risk for heart failure, and the murine model with heart failure indicated systemic insulin resistance, a state of hyperinsulinemia, developed with left ventricle pressure overload. Insulin resistance developed in the liver and visceral white adipose tissue, but cardiac insulin signaling continued to be enhanced, and excessive insulin signaling mediated detrimental effects through induction of pathological cardiac hypertrophy and ischemia in heart [151, 152]. Aging associated with an increase in cardiac insulin signaling, and this was reported as pathogenic by suppressing autophagy [153]. Insulin needs to be maintained on a physiological level, and too little or too much of this signaling is considered to become pathogenic in cardiac tissue [154, 155]. Serum IGF-1 level increased till 15 years old in both genders and thereafter declined with aging [156]. High IGF-1 predicted mortality and morbidity risk [157], and similar to insulin, studies with rodents indicated the importance of maintaining IGF-1-mediated signaling at a physiological level. IGF-1 depletion resulted in cerebro-microvascular dysfunction and deficit in spatial memory test [158]. Liver-specific depletion of IGF-1 led to dysregulation in antioxidant responses in the vasculature [159], but this model ameliorated aging-induced hepatic injury [160]. Heterozygous depletion of IGF-1 resulted in a 33% increase in lifespan in 129Sv-background mice [161]. Akt is a downstream signaling molecule for both insulin receptor and IGF-1 receptor, and constitutive activation of this molecule in cardiomyocytes enhanced cardiac hypertrophy and induced systolic dysfunction [162]. In contrast, skeletal muscle-specific overexpression of Akt induced skeletal muscle hypertrophy, together with a reduction in body weight in mice fed a high-calorie diet [163]. Insulin/IGF-1-mediated signaling has bidirectional roles and needs to be regulated in specific cells or organs to minimize detrimental effects in organs including the heart.

#### Growth hormone

Growth hormone (GH) is essential for physiological growth and has roles in lipid metabolism, body fat distribution, inflammation, and vascular health [164, 165]. Patients with GH deficiency have higher BMI and risk of dyslipidemia, therefore prone to develop hypertension and an increase in carotid intima media thickness [164, 166]. The level of GH in circulation reaches a maximum level in adolescence, and thereafter declines with aging [51]. The therapeutic potential of GH was tested in clinical trials. These showed increased muscle mass, reduction in body fat, improvement in skin elasticity, and reduction in bone demineralization, but were not associated with the enhancement of strength and functional capacity [167–171]. Issues were raised against adverse effects reported with GH administration. GH treatment is associated with carpal tunnel syndrome, arthralgias, glucose intolerance, and diabetes [168]. GH treatment was also shown to increase cancer risk [172]. For these reasons, administration of GH to individuals for the purpose of delaying aging and rejuvenation has become illegal and unethical in the USA [173].

#### Oxytocin

Oxytocin is a hormone produced mainly in the hypothalamus and secreted by the pituitary gland [174]. In humans, the level of this hormone is reduced in circulation in both genders [175, 176], and a high serum oxytocin level is positively associated with logical memory in aged females [176]. In male mice, aging led to a reduction of this hormone in plasma, and administration of oxytocin improved muscle regeneration through the activation of muscle stem cell activation and proliferation [177]. Oxytocin and two analogs reversed insulin resistance and glucose intolerance in obese mice [178]. A 4-week oxytocin treatment contributed to reducing body weight in obese humans [178]. Oxytocin also promoted liver regeneration especially in aged mice possibly through the activation of autophagic response [179]. Intranasal administration of oxytocin for 10 days was reported as safe in the aged population [180], and whether this mediates beneficial effects in individuals with cardiovascular-metabolic diseases remains an open question to be explored.

### Role of circulating steroid hormone and vitamins in age-related disorders

Circulating steroid hormones and vitamins have critical roles in the maintenance of homeostasis. In this section, we focus on the roles of steroid hormone (testosterone), vitamin D, and vitamin K.

#### Testosterone

Testosterone is the most important androgen affecting the male reproductive system. The level of testosterone reduces with chronological aging [181] and reported to show an inverse association with adverse cardiovascular outcomes, diabetes, mortality, and New York Heart Association class in heart failure [182–186]. Testosterone replacement is used in clinical settings [187]; however, in older individuals with a diminished level of this hormone, it still remains unclear whether a supply of this hormone mediates protective effect for the cardiovascular system [188]. Older men administrated with testosterone showed an increase in cardiovascular adverse events [189]. Among aged men with a reduction in circulating testosterone level, treatment with testosterone for 1 year significantly increased noncalcified plaque volume in coronary arteries [188]. Testosterone replacement therapy did not improve clinical symptoms, left ventricular ejection fraction, and N-terminal pro-B-type natriuretic peptide level in patients with heart failure [190], but another report showed this improved functional capacity and symptoms in men with advanced heart failure [191]. Administration of testosterone in aged men with low testosterone level did not lead to an improvement in memory and cognitive function in 1 year [192] and showed no benefit in vitality or walking distance [193]. Another paper reported testosterone replacement for 3 years associated with improvements in stair-climbing power, muscle mass, and power [194]. Results of clinical studies are conflicting [195], and future studies are needed to test whether replacement of this hormone becomes therapies for specific clinical conditions in selected subtypes.

#### Vitamin D

The level of vitamin D in circulation reduces with aging, and this is considered a healthcare issue to be treated [196, 197]. Vitamin D deficiency is associated with an increase in the prevalence of diabetes, hypertension, dyslipidemia, and peripheral artery disease [198]. A reduction in serum vitamin D is linked with left ventricular hypertrophy and impairment in myocardial performance [199]. Low vitamin D level is associated with dilated cardiomyopathy, and vitamin D concentration showed a negative correlation with left ventricle end-diastolic or systolic dimension [200]. Animal studies showed cardiomyocyte-specific depletion of vitamin D receptor results in cardiac hypertrophy [201]. Many clinical studies testing the therapeutic potential of vitamin D could not show a beneficial effect. Administration of vitamin D to prediabetic individuals did not reduce the risk of diabetes [202]. Vitamin D treatment did not show effects in systolic or diastolic blood pressure, physical performance, and cognitive function [203, 204]. This treatment failed to show benefit on lower extremity function and was associated with an increased risk of falls [205]. Taking oral vitamin D did not reduce blood pressure or left ventricular hypertrophy in patients with hypertension [206]. These studies indicate supplementation of vitamin D is not sufficient to mediate beneficial biological effects in humans.

#### Vitamin K

Vitamin K is a family of naphthoquinone compounds and includes vitamin K1 (phylloquinone) and K2 (menaquinone). Vitamin K1 is the predominant form in the diet. Vitamin K2 is synthesized by bacteria and included in food where bacteria are involved in the production process. Low phylloquinone level in circulation is associated with an increase in the risk of all-cause mortality [207] and is linked with higher CVD risk in aged individuals treated for hypertension [208]. Plasma phylloquinone is inversely associated with IL-6 and CRP [209], and high vitamin K is linked with a lower level of inflammatory markers and diminished risk of type 2 diabetes [210–212]. Systemic review and meta-analysis concluded vitamin K supplementation reduced vascular calcification but not vascular stiffness [213], and vitamin K1 administration was also shown to slow down the progression of aortic valve calcification [214]. Vitamin K2 treatment reduced the risk of diabetes [215], but this could not improve vascular stiffness, as analyzed with pulse wave velocity, in patients with chronic kidney disease (CKD) [216]. Another report showed vitamin K2 supplementation could not stop the progression of arterial calcification in patients with diabetes and cardiovascular disease [217].

Epigenome-wide associated study revealed a strong correlation between the epigenomic signatures of phylloquinone baseline status and response to supplementation, and further studies would show specific groups that would show a favorable response against vitamin K supplementation [218].

### Role of circulating miRNAs in age-related disorders

#### microRNA

MicroRNAs (miRs) are small non-coding RNAs produced by cells. They are engulfed in extracellular vesicles (EVs) and mediate paracrine and endocrine biological effects in local as well as remote organs. In the circulation of elderly healthy individuals, the level of miR-126-3p increased, and the concentration of this miR became low in patients with poor glycemic control [219]. Senescent human umbilical vein endothelial cells (HUVECs) were shown to secrete a high level of miR-126-3p in the conditioned medium, and authors described this miR as an active component of senescence-associated secretory phenotype (SASP) [219]. Administration of miR-126-3p mimics to ApoE^−/−^ mice suppressed atherogenesis, and antagomir-126-3p partially reversed the protective effect of 17β-estradiol in athrosclerosis [220]. Activation of miR-126-3p production was shown to play a key role in the reduction of vascular calcification [221]. The miR-30c increased with age in humans [222] and reduced in patients with coronary heart disease [223]. This miR reduced with diabetes and became lowest in coronary heart disease patients with diabetes [223]. The c-miR-21-5p level increased till 80 years old, thereafter became low in centenarians compared with 80 year-old healthy subjects, and low level of this miR is considered beneficial for longevity [224]. Several other miRNAs are reported to increase or decrease with aging or age-related diseases, and these are described in the following review articles [225, 226]. Some of these miRs would become useful as a biomarker and may become therapeutic targets with less off-target effects, and further studies are needed to test this.

### Conclusion and discussion

**Fig. 1:**
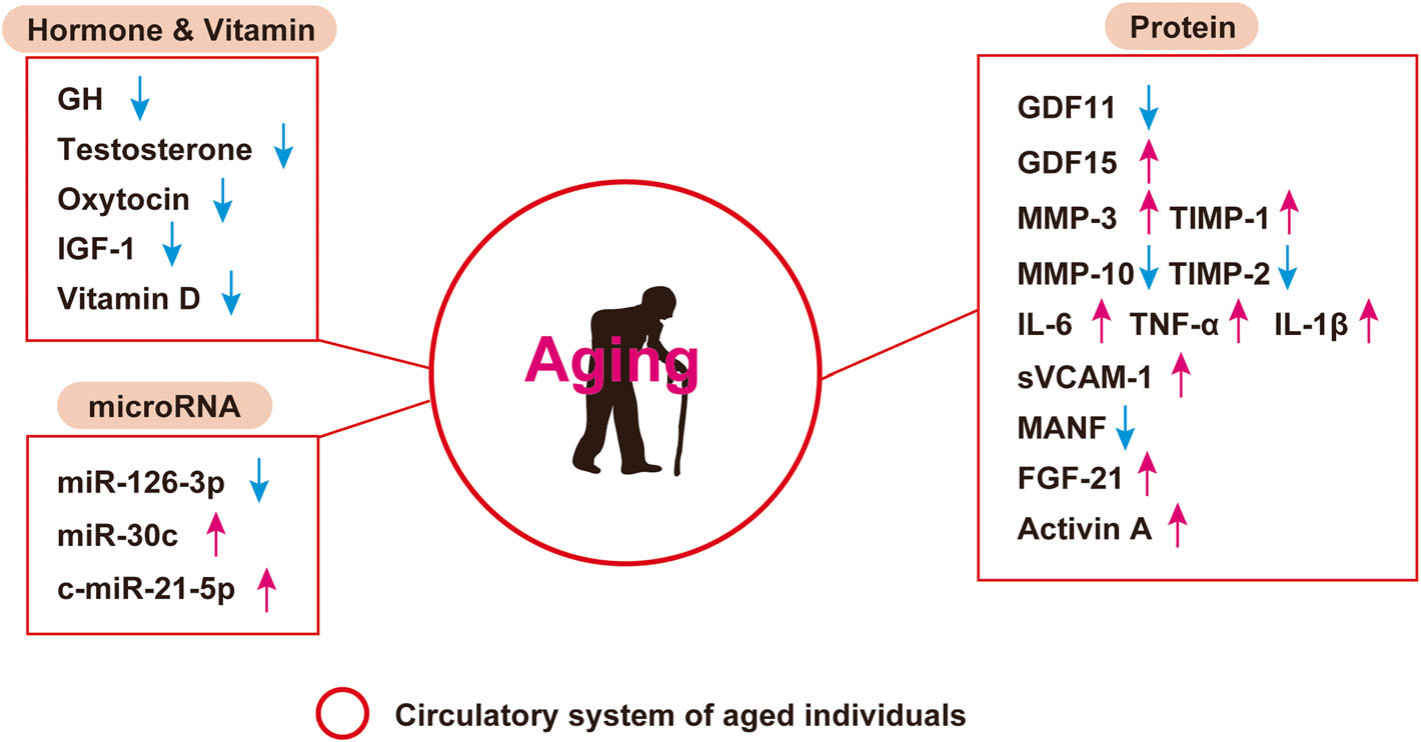
Concentration of hormones, vitamins, proteins, and microRNAs changes with chronological aging, and these are described in the figure. Growth differentiation factor (GDF), matrix metalloproteinase (MMP), tissue inhibitor of metalloproteinases (TIMP), interleukin (IL), tumor necrosis factor α (TNF-α), soluble vascular cell adhesion molecule-1(sVCAM-1), mesencephalic astrocyte-derived neurotrophic factor (MANF), fibroblast growth factor 21 (FGF-21), growth hormone (GH), insulin-like growth factor-1 (IGF-1)

**Fig. 2:**
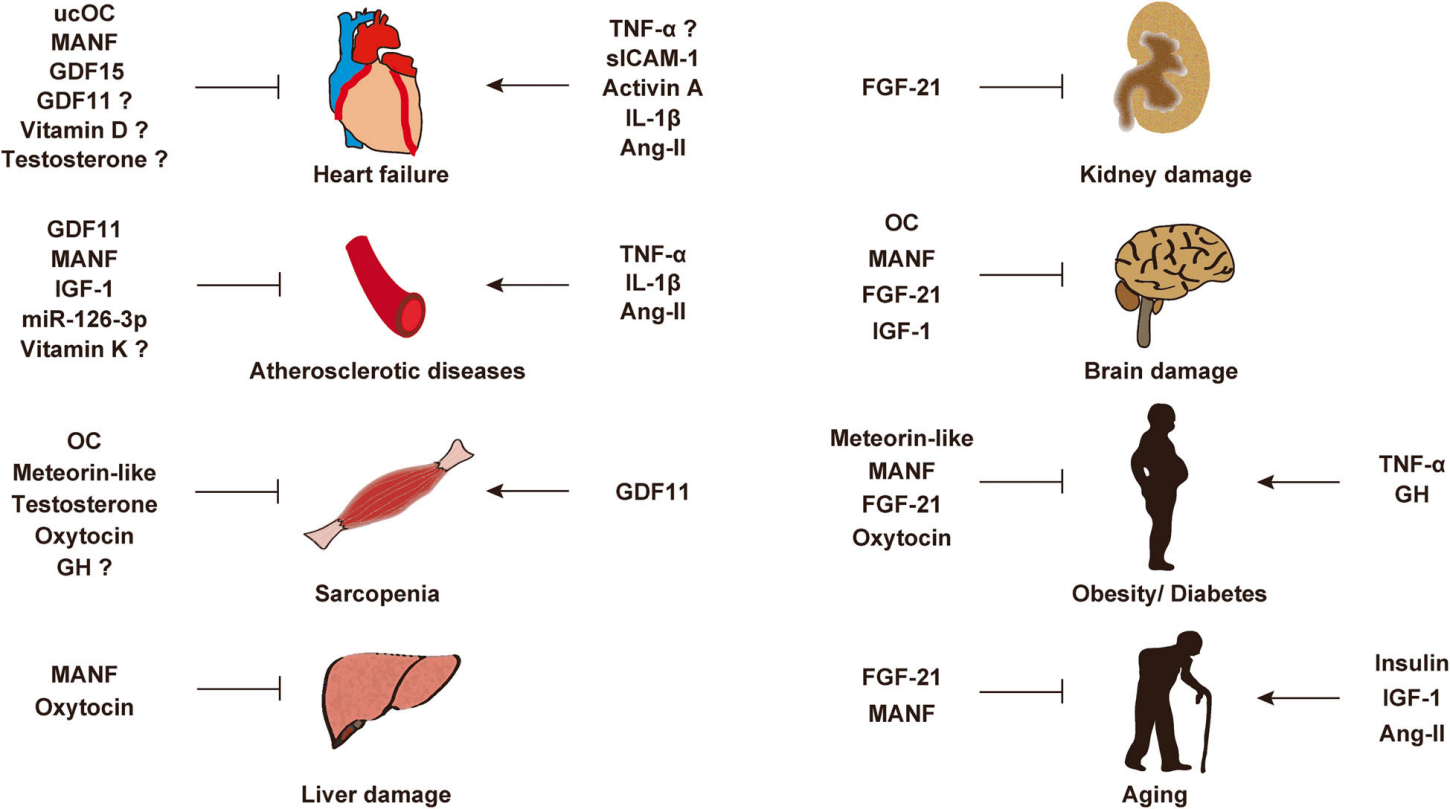
Circulating molecules characterized to suppress (inhibitory arrow) or enhance (arrow) described conditions. The question mark indicates inconsistent or controversial findings among papers. Undercarboxylated osteocalcin (ucOC), mesencephalic astrocyte-derived neurotrophic factor (MANF), growth differentiation factor (GDF), insulin-like growth factor-1 (IGF-1), osteocalcin (OC), growth hormone (GH), fibroblast growth factor 21 (FGF-21), tumor necrosis factor α (TNF-α), angiotensin II (Ang-II)

**Fig. 3:**
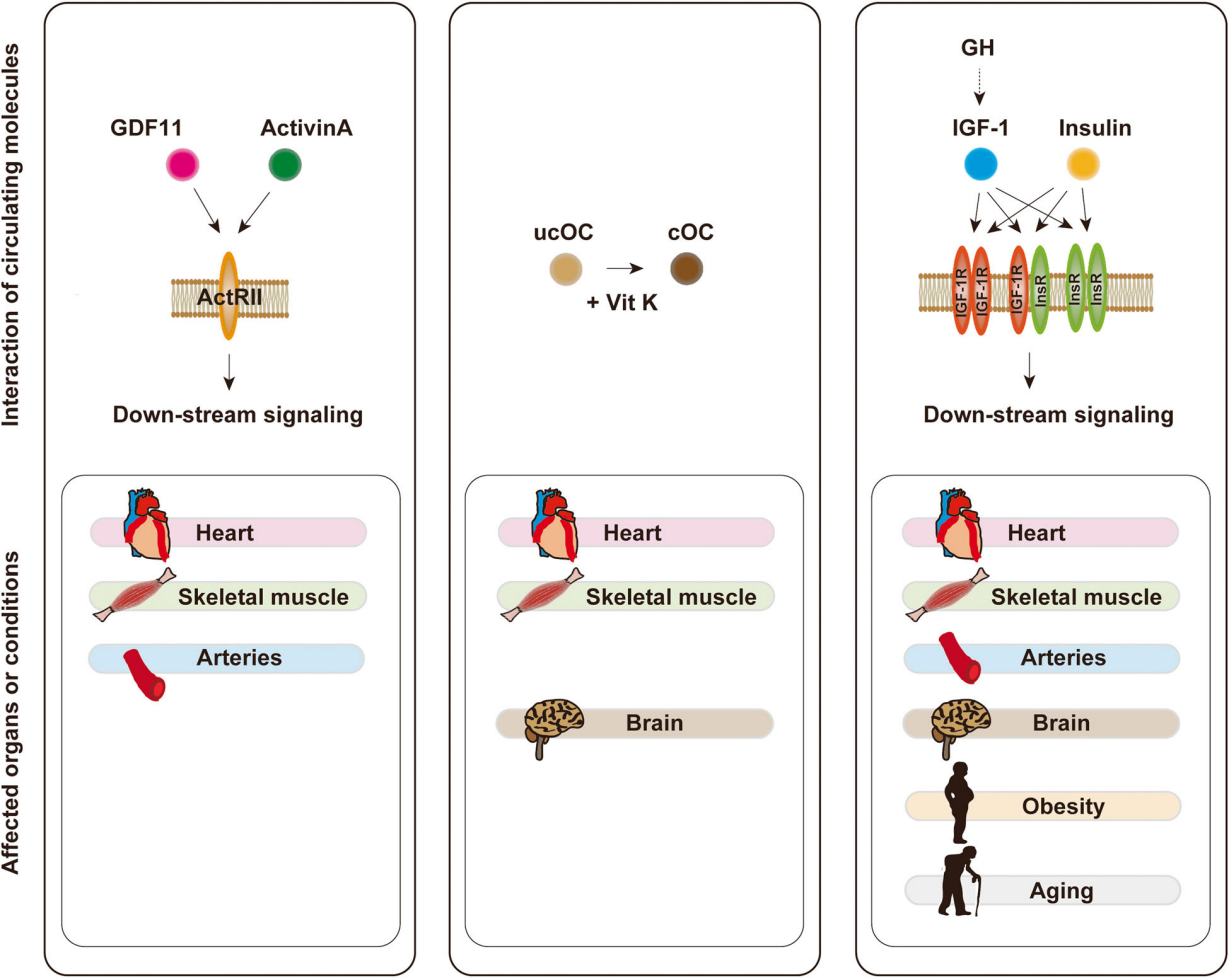
Interaction of circulating molecules described in this review article. Growth differentiation factor (GDF) 11 and activin A binds with activin type II receptor (ActRII). Vitamin K acts as a coenzyme of carboxylase and enhances the carboxylation of undercarboxylated osteocalcin (ucOC) to carboxylated osteocalcin (cOC). Growth hormone (GH) secreted from the pituitary gland increases the production of insulin-like growth factor-1 (IGF-1). Insulin and IGF-1 bind with IGF-1 receptor and/or insulin receptor (InsR). These affect organs or conditions as described in the figure

Accumulating evidence indicate exposure of aged mice to young blood results in rejuvenation. The fundamental question is whether this is applicable in humans. Given that molecules in circulation of young individuals have potential to reverse aging phenotype, the PLASMA (Plasma for Alzheimer Symptom Amelioration) study was designed for patients with Alzheimer's diseases. In this randomized clinical trial, plasma was collected from donors aged 18 to 30 years and injected into patients with Alzheimer's diseases. Outcomes concluded this procedure as safe, tolerable, and feasible [227]. In 2017, a California-based start-up company named Ambrosia began selling transfusions of young plasma for 8000USD per liter. The US Food and Drug Administration cautioned this because no proven clinical benefit has so far been demonstrated in humans, and now this start-up is completely closed [228]. This approach engulfs ethical issues, and limitation of biological materials and potential risk of transmission of known and unknown viral infection are other issues to be considered. Blood includes series of biologically active molecules such as proteins, metabolites, hormones, miRNAs, etc. In this review article, we introduced some of these fluctuate with chronological aging. They can be categorized as pro-aging or anti-aging molecules, and studies showed inhibition or activation of these molecules resulting in beneficial/detrimental/neutral effects in rodents or humans (Tables 1, 2, 3, 4, and 5; Figs. 1 and 2). Several circulating molecules are reported to interact with each other, and analyzing known or unknown interactions is essential to comprehensively understand their biological effects (Fig. 3). Levels of metabolites in circulation would also change with aging or age-related disorders [229, 230], but we did not describe these in our review article. Recent findings showed aged hematopoietic stem cells are refractory to rejuvenation through exposure to young blood [231]. Exposure to a youthful circulation did not reverse the diminished osteochondrogenic activity of skeletal stem cells [232]. These studies indicate some cells are resistant against rejuvenation. Finding mimetics of young systemic milieu, and exploration of rejuvenating factors for specific refractory cells, continues to be an interesting and important approach to find next-generation therapies for age-related disorders.

**Table 1:**
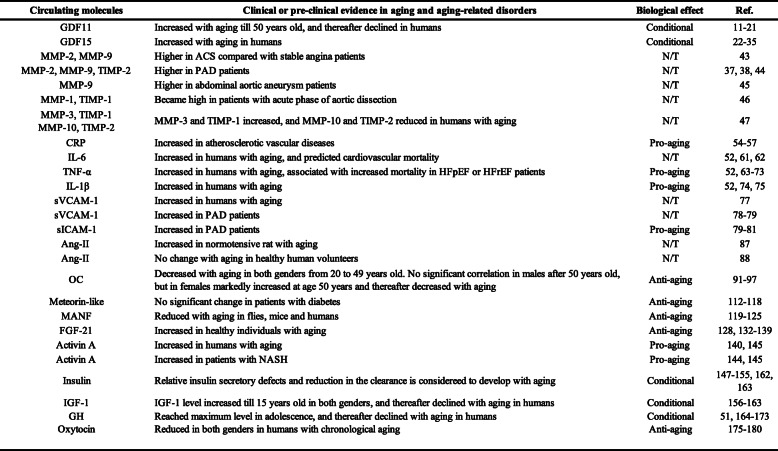
The dynamics, clinical or pre-clinical evidence, and biological effect of circulating proteins or peptides in aging and age-related disorders

**Table 2:**
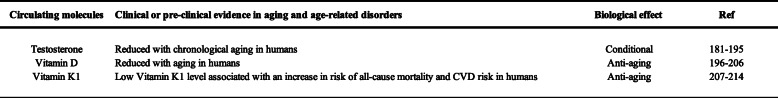
The dynamics, clinical or pre-clinical evidence, and biological effect of circulating steroid hormone and vitamins in aging and age-related disorders

**Table 3:**
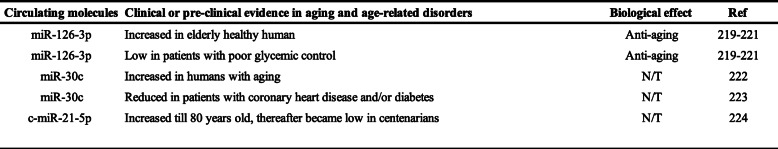
The dynamics, clinical or pre-clinical evidence, and biological effect of circulating miRNAs in aging and age-related disorders

**Table 4:**
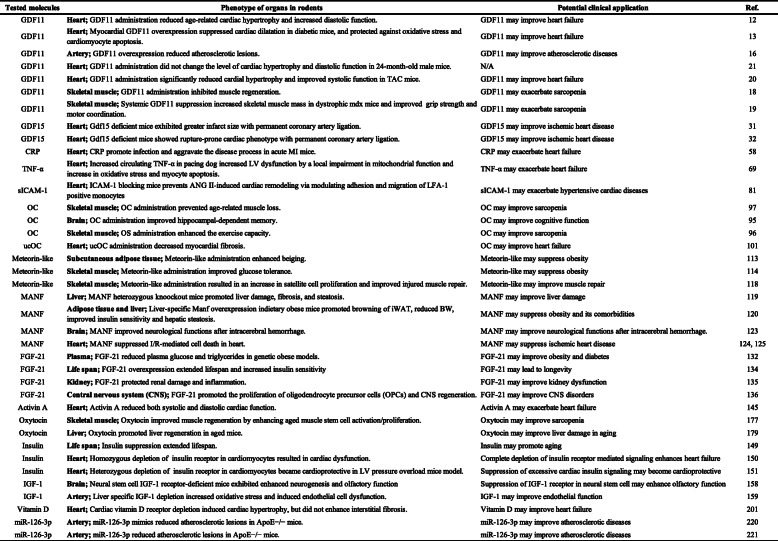
Tested molecules in circulation, phenotype of organs in rodents, and potential clinical application

**Table 5:**
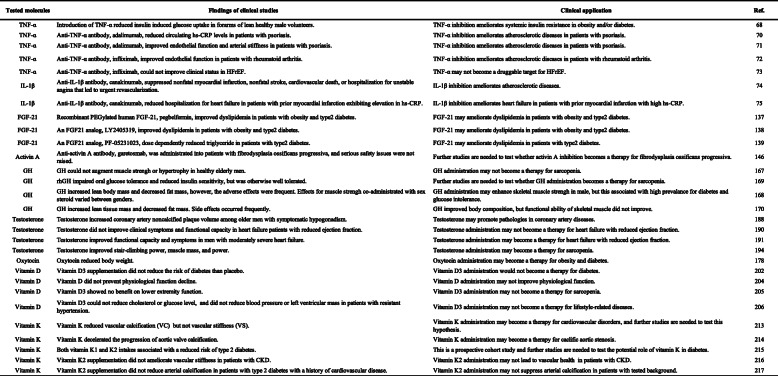
Tested molecules in circulation, findings of clinical studies, and clinical application

## Data Availability

Not applicable.
